# Maternal levels of care and association with severe maternal morbidity during birth hospitalizations

**DOI:** 10.1371/journal.pone.0353016

**Published:** 2026-07-23

**Authors:** Sara C. Handley, Molly Passarella, Joshua Radack, Ozi Amuzie, Jesse Y. Hsu, Sindhu K. Srinivas, Ciaran S. Phibbs, Scott A. Lorch

**Affiliations:** 1 Division of Neonatology, Department of Pediatrics, Children’s Hospital of Philadelphia, Philadelphia, Pennsylvania, United States of America; 2 Perelman School of Medicine, University of Pennsylvania, Philadelphia, Pennsylvania, United States of America; 3 Leonard Davis Institute of Health Economics, University of Pennsylvania, Philadelphia, Pennsylvania, United States of America; 4 Center for Clinical Epidemiology and Biostatistics, Perelman School of Medicine, University of Pennsylvania, Philadelphia, Pennsylvania, United States of America; 5 Department of Obstetrics and Gynecology, Perelman School of Medicine, University of Pennsylvania, Philadelphia, Pennsylvania, United States of America; 6 Veterans Affairs Palo Alto Health Care System, Palo Alto, California, United States of America; 7 Stanford University School of Medicine, Stanford, California, United States of America; Eulji University, KOREA, REPUBLIC OF

## Abstract

**Background:**

Maternal level of care guidelines were published to facilitate risk-appropriate care and improve outcomes, yet evidence of benefit is lacking. All birth hospitals should be equipped to provide routine childbirth care and be prepared to manage common, treatable childbirth complications. Thus, our objective was to examine the association of maternal level of care with severe maternal morbidity (SMM) without transfusion among 1) all obstetric patients and 2) obstetric patients with common, treatable childbirth complications (e.g., hemorrhage and infection).

**Methods:**

Using a retrospective observational cohort of hospital-based births, we analyzed linked vital statistic and hospital administrative data from Michigan (2010–2020), Oregon (2010–2020), Pennsylvania (2010–2018), and South Carolina (2010–2020). Maternal level of care (I [basic], II [specialty], III [subspecialty], and IV [regional perinatal health centers]) were empirically assigned using administrative data based on the care provided to patients who gave birth in each hospital annually. The primary outcome was SMM without blood product transfusion. We used modified Poisson regression to estimate adjusted risk ratios (aRRs), accounting for covariates present prior to admission for childbirth and associated with SMM.

**Results:**

Among 3,296,520 births, the SMM rate rose with increasing level (I: 0.47%, II: 0.56%, III: 0.74%, IV: 0.94%). Compared to level IV care, the SMM aRR was lower in hospitals with level I care (aRR 0.87, 95% confidence interval [CI] 0.76–0.999). Whereas, among 245,604 patients with common, treatable childbirth complications the SMM aRR was higher in hospitals with level III care (aRR 1.21, 95% CI 1.04–1.40, reference level IV).

**Conclusion:**

We found differential associations between maternal levels of care and SMM among all obstetric patients and those with common, treatable complications that arise during childbirth. While unmeasured differences in patient triage, referral, and transfer patterns may influence associations, these findings suggest differences in how hospitals of varying levels of care assess patient risk, manage complications, and perceive their capabilities, illustrating the ongoing need to improve risk-appropriate obstetric care and optimize patient outcomes.

## Introduction

Rates of maternal mortality and severe maternal morbidity (SMM) are increasing in the United States [1,2]. One approach to reduce adverse outcomes is regionalization of care, the goal of which is to improve outcomes by directing patients to hospitals with optimal capabilities for a given condition [3]. Levels of care, a fundamental aspect of regionalization, are a hospital-level characteristic that describe the intensity of care and hospital risk-based services available. The American College of Obstetricians and Gynecologists (ACOG)/Society for Maternal-Fetal Medicine (SMFM) first published maternal levels of care in 2015 and updated them in 2019 [4,5]. These levels, which are based on expert opinion, describe level I care as basic care and level IV care as regionalized, subspecialty care [4,5]. While maternal levels of care parallel neonatal levels of care and studies have consistently shown that high risk infants have better outcomes in hospitals with higher-level care, evidence demonstrating benefits of high level maternal care and obstetric outcomes is variable [6–10].

Although more than 40% of high-risk obstetric patients do not give birth in a hospital with higher level (III or IV) maternal care, published studies have not consistently identified patients for which higher level care is associated with better outcomes [7,8,10–12]. Most studies utilize hospital-reported maternal levels of care, yet data show that there are often discrepancies and over-reporting of hospital capabilities [7,8,13]. Leveraging administrative data to determine the empiric level of care based on the patients birth hospitals are providing care for offers a unique opportunity to understand the relationship between maternal levels of care and obstetric outcomes [14]. Thus, our objective was to examine if the birth hospital maternal level of care, assessed empirically, is associated with SMM during the birth hospitalization. Given the expectation that all birth hospitals should be prepared to manage common, treatable complications that arise during childbirth, a dedicated analysis of obstetric patients with hemorrhage or infection was completed with the associated hypothesis that outcomes for these patients would be similar across all levels of care.

## Methods

### Data sources

This is a retrospective, population-based observational cohort study of hospital-based live births in Michigan (2010–2020), Oregon (2010–2020), Pennsylvania (2010–2018), and South Carolina (2010–2020) [15]. These states were chosen given the availability of valid, accessible, birth parent-infant linked data, representing diverse populations, and different perinatal care systems. Vital statistics data, birth and death certificates, were linked to hospital administrative data. These data include information on all hospital discharges including hospitalization-associated International Classification of Diseases (ICD) codes. Data linkage was performed by states prior to data distribution. Using published methods, the obstetric patient-infant match rate was 94.3% with the majority of unlinked births occurring at home or at a birth center [16]. The American Hospital Association (AHA) Annual Survey data were merged with the linked data at the hospital-level to examine hospital characteristics.

Data were accessed for analyses on September 8, 2023. Prior to receipt by the research team, the data were de-identified, thus the team did not have access to data that allow for the direct identification of individuals. This study was deemed exempt by the Children’s Hospital of Philadelphia Institutional Review Board as it was classified as non-human participant research and the requirement of informed consent waived. This study followed the Strengthening the Reporting of Observational Studies in Epidemiology (STROBE) reporting guideline.

### Study population

**Fig 1:**
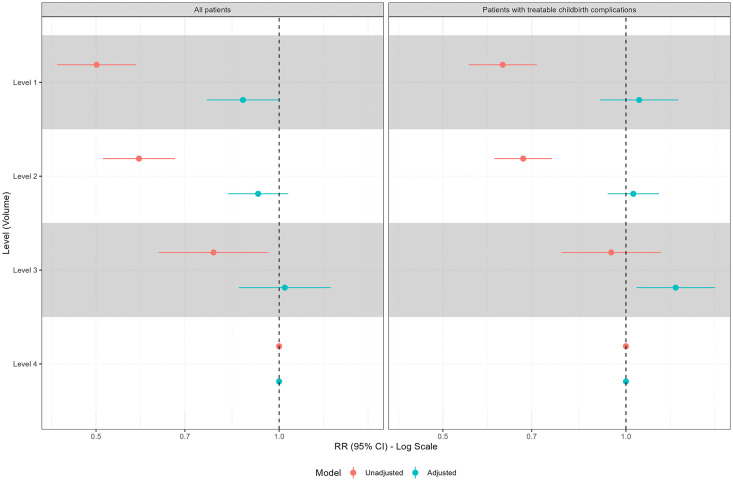
Unadjusted and Adjusted Risk Ratios of SMM by Maternal Level of Care for All Obstetric Patients (left panel) and Patients with Treatable Childbirth Complications (right panel). Figure footnote: Model covariates include birth parent age, race and ethnicity, birth parent education, birth parent insurance type, obstetric comorbidity score, parity, birth hospital location, state, and year. Abbreviation: SMM – Severe Maternal Morbidity, RR – Risk Ratio.

The included obstetric patients had linked data, with an infant born 23–44 weeks’ gestation, and birth weight 400g-8000g, as this analysis focused on birth hospitalizations associated with a viable infant. Hospital-based inclusion criteria included a hospital with obstetric services, ≥ 10 births/year, and complete location data (e.g., rural or urban based on Urban Influence Codes [UIC]) (S1 Fig 1: Cohort Identification Flow Diagram). A subpopulation of patients with the common, treatable childbirth complications of infection or hemorrhage were identified for a sub-analysis given these complications arise during childbirth and are drivers of or in the pathway to SMM (ICD codes in S1 Table).

### Study exposure, outcome, and covariates

The primary exposure was birth hospital maternal level of care. Using a published approach [14], we determined the maternal level of care empirically with ICD codes from administrative data to assign the level of care provided to obstetric patients who gave birth in each hospital each year. Based on the ACOG/SMFM level of maternal care statement and accounting for annual changes in capabilities and closures, this method is hierarchical and integrates thresholds for different types of patients to assign the level of care. The 2015 ACOG/SMFM level of care statement included examples of patients appropriate for each level of care, these descriptions were used in conjunction with Youden’s index, a summary measure of the Receiver Operating Characteristic curve to create threshold values for the annual number of each type of risk-appropriate patient [4]. The S2 Figure lists the high-risk annual patient thresholds, both number of cases and number of criteria needed for each level of care. The maternal level of care assignment is automated and output and associated flags (e.g., changes in level of care) were reviewed independently by two individuals with discussion of final assignment. This approach does not rely on hospital self-report of resources. Maternal levels of care have not been publicly reported or verified in these four states, limiting external validation of levels.

The primary outcome was SMM during the birth hospitalization, which was determined from the hospital discharge status. The SMM definition applied is published by the Maternal and Child Health Bureau (codes listed in S2 Table) [17]. As proposed in the literature, the primary outcome excluded indicators for blood product transfusion given the disproportionate contribution to SMM and inconsistent coding of transfusions [18]. A secondary analysis examined the outcome SMM including blood product transfusion.

Based on prior literature, study covariates examined included: age (<20, 20–24, 25–35, 36–40, and >40 years) [19], race and ethnicity (Hispanic, non-Hispanic Asian, non-Hispanic-Black, non-Hispanic Other, and non-Hispanic White) as a proxy variable for structural racism [20], insurance type (government, private, self-pay, other), education attainment (no high school, some high school, high school degree, some college, 4 years of college, > 4 years of college, missing) [21], obstetric comorbidity index score [22], parity (nulliparous/multiparous) [23], mode of birth (vaginal/cesarean) [24], the two treatable childbirth complications (infection and hemorrhage), birth hospital location (based on UIC; metropolitan [UIC 1, 2], micropolitan [UIC 3,5,8], non-core [UIC 4,6,7,9,10,11,12]) [25], state [26], and birth year [27]. Described by Leonard et al, the obstetric comorbidity index score captures 26 different pre-existing and pregnancy-associated medical conditions [22]. We used the obstetric comorbidity index score without blood product transfusion to capture patient risk. The AHA annual survey includes the hospital characteristics of obstetric volume (calculated from the number of births annually). hospital ownership, and teaching status.

### Data analysis

Obstetric patient characteristics were compared by maternal level of care using Analysis of Variance, Kruskal-Wallis, or chi-square tests based on data type. Hospital characteristics by level of care were examined. We performed modified Poisson regression models examining level of care with SMM to report unadjusted and adjusted risk ratios (RR and aRR, respectively). Models accounted for covariates previously associated with SMM in the literature and present prior to admission for childbirth with robust standard errors clustering for hospital. Analyses were completed for all obstetric patients, followed by a sub-analysis of patients with infection or hemorrhage. We conducted a sensitivity analysis examining temporal heterogeneity before and after publication of the first maternal level of care guidelines in 2015 (2010–2015 and 2016–2020). We also conducted two secondary analyses assessing the association of maternal levels of care with SMM including blood product transfusion and stratification by state given variation in state-based health systems, approaches to regionalization, and statewide quality improvement initiatives. Data were managed in SAS version 9.4 and analyzed in Stata version 17.

## Results

Of 3,296,520 obstetric patients, 13.8% gave birth in a hospital with level I care, 32.6% with level II care, 9.1% with level III care, and 44.5% with level IV care. Table 1 shows patient characteristics by level of care. There were 245,604 (7.5%) patients identified with infection or hemorrhage during the birth hospitalization (patient characteristics in S3 Table). Hospitals with higher level maternal care were often non-profit, teaching hospitals, with higher annual birth volume (Table 1).

**Table 1:** Patient-level Characteristics by Maternal Level of Care (N = 3,296,520).

Characteristic	Maternal Level of Care				P-value
	I n (%)	II n (%)	III n (%)	IV n (%)	
	456,541 (13.8)	1,073,982 (32.6)	300,264 (9.1)	1,465,733 (44.5)	
Birth Parent Age (years)					<0.001
<20	37,641 (8.2)	67,456 (6.3)	17,917 (6.0)	85,734 (5.9)	
20-24	125,549 (27.5)	240,656 (22.4)	61,644 (20.5)	294,088 (20.1)	
25-34	244,258 (53.5)	615,078 (57.3)	173,088 (57.7)	845,524 (57.7)	
35-39	40,788 (8.9)	125,566 (11.7)	38,949 (13.0)	196,589 (13.4)	
>40	8,305 (1.8)	25,226 (2.4)	8,666 (2.9)	43,798 (3.0)	
Birth Parent Race and Ethnicity					<0.001
Hispanic	41,692 (9.1)	80,920 (7.5)	23,799 (7.9)	152,614 (10.4)	
Non-Hispanic Asian	6,507 (1.4)	36,498 (3.4)	9,592 (3.2)	66,633 (4.6)	
Non-Hispanic Black	31,001 (6.8)	151,241 (14.1)	64,937 (21.6)	313,568 (21.4)	
Non-Hispanic Other	20,709 (4.5)	44,197 (4.1)	12,810 (4.3)	59,796 (4.1)	
Non-Hispanic White	356,632 (78.1)	761,126 (70.9)	189,126 (63.0)	873,122 (59.6)	
Birth Parent Education Attainment					<0.001
No High School	10,628 (2.3)	19,809 (1.8)	4,790 (1.6)	31,710 (2.2)	
Some High School	59,971 (13.1)	106,124 (9.9)	28,259 (9.4)	151,723 (10.4)	
High School Degree	142,518 (31.2)	270,496 (25.2)	72,160 (24.0)	342,627 (23.4)	
Some College	152,645 (33.4)	347,240 (32.3)	93,676 (31.2)	423,384 (28.9)	
4 Years College	61,328 (13.4)	208,242 (19.4)	61,626 (20.5)	304,041 (20.7)	
>4 Years College	28,098 (6.2)	117,910 (11.0)	38,464 (12.8)	201,173 (13.7)	
Missing	1,353 (0.30)	4,161 (0.39)	1,289 (0.43)	11,075 (0.76)	
Birth Parent Insurance					<0.001
Government	232,987 (51.0)	459,218 (42.8)	130,410 (43.4)	635,026 (43.3)	
Private	209,493 (45.9)	580,153 (54.0)	164,470 (54.8)	803,085 (54.8)	
Self-Pay	7,586 (1.7)	12,982 (1.2)	1,863 (0.62)	13,963 (0.95)	
Other	6,475 (1.4)	21,629 (2.0)	3,521 (1.2)	13,659 (0.93)	
Obstetric Comorbidity Score (Non-Transfusion), mean (SD)	4.10 (7.12)	4.79 (8.12)	5.64 (9.34)	7.69 (11.31)	<0.001
Nulliparous	175,270 (38.4)	429,794 (40.0)	119,108 (39.7)	586,022 (40.0)	<0.001
Cesarean birth	140,177 (30.7)	341,541 (31.8)	99,985 (33.3)	485,321 (33.1)	<0.001
Patients with treatable complications	27,761 (6.1)	67,699 (6.3)	18,873 (6.3)	131,271 (9.0)	<0.001
Infection	13,501 (3.0)	35,584 (3.3)	10,490 (3.5)	66,658 (4.6)	<0.001
Hemorrhage	14,823 (3.3)	33,608 (3.1)	8,815 (2.9)	68,879 (4.7)	<0.001
SMM (without blood product transfusion)	2,160 (0.47)	5,965 (0.56)	2,211 (0.74)	13,836 (0.94)	<0.001
SMM (including blood product transfusion)	6,576 (1.4)	14,852 (1.4)	4,453 (1.5)	28,321 (1.9)	<0.001
Birth Hospital Location^a^					<0.001
Metropolitan	228,479 (50.1)	995,674 (92.7)	285,784 (95.2)	1,465,733 (100)	
Micropolitan	165,195 (36.2)	76,101 (7.1)	14,480 (4.8)	0 (0.0)	
Noncore	62,867 (13.8)	2,207 (0.2)	0 (0.0)	0 (0.0)	
Annual Obstetric Volume					<0.001
10-500	272,124 (59.6)	12,609 (1.2)	0 (0.0)	0 (0.0)	
501-1000	179,047 (39.2)	316,437 (29.5)	0 (0.0)	3,229 (0.2)	
1001-2000	5,370 (1.2)	614,461 (57.2)	119,534 (39.8)	104,837 (7.2)	
>2000	0 (0.0)	130,475 (12.2)	180,730 (60.2)	1,357,667 (92.6)	
Hospital Ownership					<0.001
Government	34,479 (7.6)	43,638 (4.1)	0 (0.0)	144,560 (9.9)	
Non-Profit	379,372 (83.1)	863,097 (80.4)	260,392 (86.7)	1,277,235 (87.1)	
Profit	42,390 (9.3)	163,316 (15.2)	39,872 (13.3)	43,938 (3.0)	
Missing	300 (0.07)	3,931 (0.4)	0 (0.0)	0 (0.0)	
Teaching Status^b^					<0.001
Major-Teaching	7,497 (1.6)	29,220 (2.7)	84,550 (28.2)	769,983 (52.5)	
Minor-Teaching	125,477 (27.5)	636,464 (59.3)	160,528 (53.5)	619,732 (42.3)	
Non-Teaching	323,201 (70.8)	404,367 (37.7)	55,186 (18.4)	76,018 (5.2)	
Missing	366 (0.1)	3,931 (0.4)	0 (0.0)	0 (0.0)	

Abbreviations: SD – Standard Deviation, SMM – Severe Maternal Morbidity

^a^Rurality is defined by Urban Influence Codes [UIC] with metropolitan: UIC 1, 2; micropolitan: UIC 3,5,8; non-core: UIC 4,6,7,9,10,11,12.

^b^Teaching status: Major teaching is defined by the American Hospital Association as being a member of the Council of Teaching Hospitals. Minor teaching is defined as those that are teaching hospitals but are not members and have at least one intern or resident.

Among all obstetric patients, the proportion of patients with SMM increased with increasing level of care; level I: 2,160 (0.47%), level II: 5,965 (0.56%), level III: 2,211 (0.74%), and level IV: 13,836 (0.94%) (Table 1). Similarly, the unadjusted models examining the association of level of care (reference level IV) with SMM found a lower risk ratio (RR) of SMM in levels I, II, and III (Fig 1). In multivariable analysis, this association remained significant only for hospitals with level I care (adjusted risk ratio [aRR] 0.87, 95% confidence interval [CI] 0.76, 0.999) (Fig 1, full model output in S1 File). The sensitivity analysis examining associations during the pre-guideline (2010–2015) and post-guideline (2016–2020) epochs among all obstetric patients found no associations between maternal level of care and SMM in the pre-guideline epoch (level I aRR 0.91, 95% CI 0.77, 1.07; level II aRR 0.99, 95% CI 0.86, 1.13; level III aRR 1.13, 95% CI 0.91, 1.40; level IV reference). In the post-guideline epoch there was a lower aRR of SMM in hospitals with level I and level II maternal care among all obstetric patients (level I aRR 0.83, 95% CI 0.71, 0.97; level II aRR 0.84, 95% CI 0.74, 0.95; level III aRR 0.87, 95% CI 0.74, 1.02; level IV reference).

Among patients with a treatable childbirth complication the SMM pattern was consistent (level I: 634 [2.3%], level II: 1,671 [2.5%], level III 650 [3.4%], and level IV 4,780 [3.6%] (S3 Table). Yet, in this sub-population, the aRR of SMM was increased in hospitals with level III care (aRR 1.21, 95% CI 1.04, 1.40, reference level IV) (Fig 1, full model output in S2 File). When stratifying by complication, the adjusted models examining patients with infection showed a significant association between level II care and a decreased risk ratio of SMM (aRR 0.85, 95% CI 0.74, 0.97; Table 2, full model output in S3 File). Whereas among patients with hemorrhage, the risk ratio of SMM was significantly increased for both level II and level III care, 1.17 and 1.24 times higher respectively than births in hospitals with level IV care (Table 2, full model output in S4 File).

**Table 2:** Unadjusted and Adjusted Risk Ratios of SMM by Maternal Level of Care Stratified by Common Treatable Childbirth Complication.

Maternal Level of Care	Infection		Hemorrhage	
	RR (95% CI)	aRR (95% CI)	RR (95% CI)	aRR (95% CI)
Level I	0.58 (0.48, 0.69)	0.90 (0.75, 1.08)	0.65 (0.55, 0.77)	1.19 (0.99, 1.42)
Level II	0.57 (0.49, 0.66)	0.85 (0.74, 0.97)	0.75 (0.66, 0.87)	1.17 (1.04, 1.30)
Level III	0.88 (0.74, 1.06)	1.15 (0.95, 1.39)	1.04 (0.82, 1.32)	1.24 (1.06, 1.46)
Level IV	Reference	Reference	Reference	Reference

Abbreviations: SMM – Severe Maternal Morbidity, RR –Risk Ratio, aRR – Adjusted Risk Ratio, CI – Confidence Interval

Model covariates include birth parent age, race and ethnicity, birth parent education, birth parent insurance type, obstetric comorbidity score, parity, birth hospital location, state, and year.

In the secondary analysis examining the outcome of SMM including blood product transfusion we found no associations of maternal levels of care with this outcome in the adjusted models including all obstetric patients (S4 Table). Among those with common treatable childbirth conditions, we found a higher aRR of SMM including blood product transfusion in hospitals with level I maternal care (S4 Table). This finding reflects the higher aRR among the subset of obstetric patients who experienced hemorrhage (level I aRR 1.34, 95% 1.10, 1.64; reference level IV) and was noted to a lesser degree in hospitals with level II maternal care (aRR 1.18, 95% CI 1.002, 1.40; reference level IV) (S4 Table).

The secondary analysis stratified by state, which examined the primary outcome (SMM without blood product transfusion), are shown are point estimate ranges in S5 Table. Though point estimates varied across states, patterns generally mirror the primary findings with point estimates <1 among all obstetric patients in hospitals with level I care (reference level IV) and point estimates >1 among obstetric patients with common treatable childbirth conditions, both infection and hemorrhage, in hospitals with level III care (reference level IV).

## Discussion

In this four-state cohort, the aRR of SMM for all obstetric patients was lower in birth hospitals with level I care, compared to level IV care. When examining patients with common, treatable childbirth complications that should be appropriately managed by all birth hospitals regardless of level of care, the risk ratio of SMM was higher in level III hospitals, contrary to our hypothesis that rates would be similar. These associations may suggest differences in hospitals and how hospitals approach and facilitate risk-appropriate care during childbirth, with respect to screening, triage, transfer, and their own capabilities.

In this study, obstetric patients who gave birth in hospitals with level I care, which is intended to provide care to lower risk patients and to effectively detect, stabilize, and initiate appropriate intervention when unexpected problems occur [5], had a lower risk of SMM. While this finding was present in the cohort as a whole, it was more prominent (lower point estimates and a similar finding in hospitals with level II maternal care) in the post-guideline epoch. Many obstetric patients are lower-risk, and these associations support risk-appropriate care for this population. Even among the lowest-risk patients complications that require non-routine care still arise [28], and in our data 0.47% of births in level I hospitals developed a SMM. A plausible hypothesis may be that after publication of the 2015 level of care guidelines hospitals with lower level care thought more about their resources and potential limitations and augmented approaches to screening, triage, and transport of obstetric patients at higher risk of complications. Obstetric patient referral and transport peri-delivery is complicated by a variety of factors that could be contributing to findings, including birth hospital choice, self-referral, in-office referral, transfer by personal car (vs ambulance), and hospital-to-hospital intrapartum transport networks, only the latter of which is captured in administrative data. While some characteristics of hospitals with level I care and surrounding systems may be unmeasured, they appear to be facilitating risk-appropriate care for low-risk obstetric patients over time [29].

Conversely, among patients who developed a common complication during childbirth the rate of SMM was highest in hospitals with level III care, which provide subspecialty care and have consistent access to obstetricians, intensive care units, advanced imaging, and basic interventional radiology services [5]. Hospitals with level III and IV care differ with respect to patient case mix and hospital capabilities (on call versus 24-hour in-hospital subspecialists) and delineating these differences in administrative data is challenging. Our analysis could not account for clinical acuity at admission, timing of intrapartum deterioration, indication for emergency cesarean birth (if indicated), severity of infection or hemorrhage, or transfer dynamics (e.g., a patient became too unstable to transfer to a hospital with level IV care). Interestingly, findings differed when stratified by complication type, for patients with infection, the risk of SMM was lower in level II hospitals, while for those with hemorrhage, the risk of SMM was higher in level II and III hospitals. When considering the secondary analysis of SMM including blood product transfusion, there were no detectable differences between levels of care for patients with infection, while the risk of SMM was significantly higher for patients with hemorrhage in level I and II hospitals. Blood product transfusion is a common treatment for hemorrhage. In hospitals with lower level maternal care where other resources or services to manage hemorrhage may not be available, thresholds to transfuse may be lower than level III or IV hospitals, which could contribute to these findings. Collectively, these findings support further nuanced analysis with respect to patient acuity and ongoing studies of practices surrounding hemorrhage management.

Unlike prior studies of levels of care, which often include dedicated analyses of high-risk obstetric patients [7,10,12], this study focused on common, treatable childbirth complications. While all birth hospitals do not have the same patient case mix and are not expected to have the expertise and resources to care for complex obstetric patients, the ability to quickly recognize and appropriately respond to patients with hemorrhage or infection is fundamental to peripartum care. By definition, lower-level hospitals and their patients differ from higher-level hospitals. For example, level IV hospitals are regional perinatal centers that should be engaging in educational initiatives, outcome review, and quality improvement (QI) initiatives with surrounding lower-level hospitals. Level IV centers may have completed more QI work or implemented robust hemorrhage protocols. Engagement in QI, particularly for postpartum hemorrhage, has been associated with marked improvement in outcomes [30] and partnership to disseminate targeted educational materials and facilitate QI efforts may help mitigate the risk of SMM among patients with common childbirth complications in hospitals with lower level care.

While further evaluation of maternal levels of care and risk-appropriate obstetric care during childbirth are needed, these findings bring attention to this important topic. Considering potential future avenues to optimize risk-appropriate care for obstetric patients in the United States could include standardizing state level of care guidelines, incentivizing and expanding level of care verification programs, which ACOG has made efforts to expand in partnership with The Joint Commission [31,32], and increasing visibility and public reporting of level of care data and associated hospital obstetric capabilities. Systematic changes that optimize risk-appropriate care for obstetric patients may improve outcomes.

### Study limitations

This analysis has limitations. First, verified sources for maternal levels of care are unavailable, making exposure misclassification difficult to evaluate. However, most prior approaches rely on versions of hospital self-report, which are often biased by overreporting capabilities and may be a disservice to patients. For example, a hospital that reports level III capabilities, but provides services consistent with level II care, will be evaluated as a level II hospital in this study, more likely reflecting their true capabilities. Second, unmeasured confounding is present in observational studies and while we integrated the obstetric comorbidity index, there may be additional unmeasured factors at the patient or hospital-level not included in this analysis, especially for the highest-level (III and IV) hospitals with the most acute patients. At the patient level, administrative data lack the granularity to determine temporality (e.g., illness acuity on admission, timing of decompensation relative to progression of labor or time of birth, indications for an emergency cesarean birth, the severity of infection or hemorrhage). At the hospital-level determining the specifics of hospital resources with respect to off-site/on-call versus on-site/immediately available is limited. Collectively, unmeasured factors may influence the associations detected in hospitals with level I and level III maternal care. Third, the data from these four states lack a consistent maternal identifier, which limits clustering at the patient-level (for those with more than one birth). However, our models clustered at the hospital-level, which produces more conservative results (e.g., wider confidence intervals) compared to patient-level clustering. Fourth, the sensitivity analysis examining temporal heterogeneity revealed differences between the pre- and post-guideline epochs. While this trend could reflect changes in obstetric care organization that optimize risk-appropriate care, it may also be influenced by the evolving rates of SMM over time, uncoordinated rollout of state-wide QI initiatives, other changing policies, lack of available 2019−2020 PA data, or the transition to ICD-10 codes. Fifth, this is a population-based analysis of four states with varied health systems, populations, and geography and while our secondary analysis showed similar significance patterns across these four states, other states may show different effects.

## Conclusion

In the four states studied, compared to level IV care, we found associations of level I care with lower rates of SMM among all obstetric patients and level III care with higher rates of SMM among patients with common treatable complications during childbirth. While unmeasured differences in patient triage, referral, and transfer patterns may influence associations, these data illustrate the ongoing need to understand and refine approaches to risk-appropriate obstetric care delivery. These findings can inform future studies that evaluate hospital and health system-based efforts to optimize patient risk and hospital capability assessment and operationalize maternal levels of care to ultimately improve patient outcomes.

## Supporting information

S1 Fig Cohort Identification Flow Diagram. (TIF)

S2 Fig Approach to Identifying Maternal Levels of Care. (TIF)

S1 Table Codes Used to Identify Obstetric Patients with Common Treatable Childbirth Complications. (DOCX)

S2 Table Codes Used to Identify Obstetric Patients with Severe Maternal Morbidity. (DOCX)

S3 Table Characteristics Among Obstetric Patients with a Common Treatable Childbirth Complications by Maternal Level of Care (N = 245,604). (DOCX)

S4 Table Associations of levels of maternal care with SMM including blood product transfusion. (DOCX)

S5 Table Supplemental analysis stratified by state reporting range of point estimates from the adjusted models examining the association of level of maternal care and SMM without blood product transfusion among 1) all obstetric patients, 2) those with common treatable childbirth complications, 3) those with infection, and 4) those with hemorrhage. (DOCX)

S1 File Full Model for association between level of maternal care and SMM without transfusion for all obstetric patients. (DOCX)

S2 File Full Model for association between level of maternal care and SMM without transfusion for obstetric patients with common treatable childbirth complications. (DOCX)

S3 File Full Model for association between level of maternal care and SMM without transfusion for obstetric patients with infection. (DOCX)

S4 File Full Model for association between level of maternal care and SMM without transfusion for obstetric patients with hemorrhage. (DOCX)
