# Supplementary material for: Maternal levels of care and association with severe maternal morbidity during birth hospitalizations
Source: PLoS One. 2026 Jul 23;21(7):e0353016. doi: 10.1371/journal.pone.0353016 (PMC13395347; doi:10.1371/journal.pone.0353016)
Supplement: S1 Table — (DOCX) [file pone.0353016.s003.docx]

**S1 Table. Codes Used to Identify Obstetric Patients with Common Treatable Childbirth Complications**

| Condition | ICD-9 Codes | ICD-10 Codes |
| --- | --- | --- |
| Infection | 481, 485, 486, 541, 542, 566, 575.0, 575.12, 590.2, 728.86, 042.X, 070.X, 480.X, 482.X, 483.X, 484.X, 487.X, 488.X, 540.X, 567.X, 590.0X, 590.1X, 647.3X, 647.6X, 647.8X, 647.9X, 670.0X, 670.1X, 670.3X, 670.4X | B15.X, B16X, B17.X, B18.X, B19.X, B20.X, J09.X, J10.X, J11.X, J12.X, J13.X, J14.X, J15.X, J16.X, J17.X, J18.X, K35.X, K36, K36, K61.X, K81.0, K81.2, M72.6, N10, N11, N15.1, O86.12, O86.21, O86.81, O98.1X, O98. 4X, O98.5X, O98.7X, O98.8X, O98.9X |
| Hemorrhage | 666 (excluding 666.3) | O72 (excluding O72.3) |

Excluded codes are included in the outcome of severe maternal morbidity via diagnosis of disseminated intravascular coagulation
