# Supplementary material for: Maternal levels of care and association with severe maternal morbidity during birth hospitalizations
Source: PLoS One. 2026 Jul 23;21(7):e0353016. doi: 10.1371/journal.pone.0353016 (PMC13395347; doi:10.1371/journal.pone.0353016)
Supplement: S2 Table — (DOCX) [file pone.0353016.s004.docx]

**S2 Table. Codes Used to Identify Obstetric Patients with Severe Maternal Morbidity**

| Condition Type | ICD-9 Codes | ICD-10 Codes |
| --- | --- | --- |
| Acute Myocardial Infarction | 410 | I21, I22 |
| Aneurysm | 441 | I71, I79.0 |
| Acute Renal Failure | 584.5, 584.6, 584.7, 584.8, 584.9, 669.3 | N17, O90.4 |
| Acute Respiratory Distress | 518.5, 518.81, 518.82, 518.84, 799.1 | J80, J95.1, J95.2, J95.3, J95.82, J96.0, J96.2, J96.9, R0603, R092 |
| Amniotic Fluid Embolism | 673.1 | O88.112, O88.113, O88.119, O88.12, O88.13 |
| Cardiac Arrest/Ventricular Fibrillation | 427.41, 427.42, 427.5 | I46, I490 |
| Disseminated Intravascular Coagulation | 286.6, 286.9, 614.3, 666.3 | D65, D68.8, D68.9, O45.002, O45.003, O45.009, O45.012, O45.013, O45.019, O45.022, O45.023, O45.029, O45.092, O45.093, O45.099, O46.002, O46.003, O46.009, O46.012, O46.013, O46.019, O46.022, O46.023, O46.029, O46.092, O46.093, O46.099, O67.0, O72.3 |
| Eclampsia | 642.6 | O15 |
| Heart Failure/Arrest During Procedure | 997.1 | I97.12, I97.13, I97.71 |
| Puerperal Cerebrovascular Disorders | 046.3, 348.39, 362.34, 430, 431, 432, 433, 434, 435, 436 437, 671.5, 674.0, 997.02 | A81.2, G45, G46, G93.49, H34.0, I60, I61, I62, I63, I65, I66, I67, I68, O22.50, O22.52, O22.53, I97.81, I97.82, O87.3 |
| Pulmonary Edema | 518.4 | J81.0 |
| Acute Heart Failure | 428.0, 428.1, 428.20, 428.21, 428.23, 428.30, 428.31, 428.33, 428.40, 428.41, 428.43, 428.9 | I50.1, I50.20, I50.21, I50.23, I50.30, I50.31, I50.33, I50.40, I50.41, I50.43, I50.810, I50.811, I50.813, I50.814, I50.82, I50.83, I50.84, I50.89, I50.9 |
| Severe Anesthesia Complications | 668.0, 668.1, 668.2, 995.4, 995.86 | O29.112, O29.113, O29.119, O29.122, O29.123, O29.129, O29.192, O29.193, O29.199, O29.212, O29.213, O29.219, O29.292, O29.293, O29.299,  O74.0, O74.1, O74.2, O74.3, O89.01, O89.09, O89.1, O89.2, T88.2XXA, T88.3XXA |
| Sepsis | 038, 44.9, 670.2, 785.52, 995.91, 995.92, 998.02 | A32.7, A40, A41, I76, O85, O86.04, R65.20, R65.21, T81.12XA, T81.44XA |
| Shock | 669.1, 785.50, 785.51, 785.59, 995.0, 998.0, 998.00, 998.01, 998.09 | O75.1, R57, T78.2XXA, T81.10XA, T81.11XA, T81.19XA, T88.6XXA |
| Sickle Cell Disease | 282.42, 282.62, 282.64, 282.69, 289.52 | D57.0, D57.21, D57.41, D57.81 |
| Air and Thrombotic Embolism | 415.0, 415.1, 673.0, 673.2, 673.3, 673.8 | I26.01, I26.02, I26.09, I26.90, I26.92, I26.93, I26.94, I2.699, O88.012, O88.013, O88.019, O88.02, O88.03, O88.212, O88.213, O88.219, O88.22, O88.23, O88.312, O88.313, O88.319, O88.32, O88.33, O88.812, O88.813, O88.819, O88.82, O88.83, T80.0XXA |
