# Supplementary material for: Maternal levels of care and association with severe maternal morbidity during birth hospitalizations
Source: PLoS One. 2026 Jul 23;21(7):e0353016. doi: 10.1371/journal.pone.0353016 (PMC13395347; doi:10.1371/journal.pone.0353016)
Supplement: S3 Table — (DOCX) [file pone.0353016.s005.docx]

**S3 Table. Characteristics Among Obstetric Patients with a Common Treatable Childbirth Complications by Maternal Level of Care (N=245,604)**

|  | Maternal Level of Care | | | |  |
| --- | --- | --- | --- | --- | --- |
| Characteristics | 1  n (%) | 2  n (%) | 3  n (%) | 4  n (%) | p-value |
|  | 27,761 (11.3) | 67,699 (27.6) | 18,873 (7.7) | 131,271 (53.4) |  |
| Age (years) |  |  |  |  | <0.001 |
| <20 | 2,482 (8.9) | 4,502 (6.7) | 1,104 (5.9) | 7,889 (6.0) |  |
| 20-24 | 7,735 (27.9) | 15,951 (23.6) | 4,004 (21.2) | 27,631 (21.1) |  |
| 25-34 | 14,601 (52.6) | 37,590 (55.5) | 10,560 (56.0) | 73,110 (55.7) |  |
| 35-39 | 2,397 (8.6) | 7,908 (11.7) | 2,581 (13.7) | 18,008 (13.7) |  |
| >40 | 546 (2.0) | 1,748 (2.6) | 624 (3.3) | 4,633 (3.5) |  |
| Race and Ethnicity |  |  |  |  | <0.001 |
| Hispanic | 3,119 (11.2) | 6,081 (9.0) | 1,438 (7.6) | 14,706 (11.2) |  |
| Non-Hispanic Asian | 466 (1.7) | 2,444 (3.6) | 552 (2.9) | 6,687 (5.1) |  |
| Non-Hispanic Black | 1,807 (6.5) | 11,822 (17.5) | 5,587 (29.6) | 34,357 (26.2) |  |
| Non-Hispanic Other | 1,532 (5.5) | 3,058 (4.5) | 847 (4.5) | 5,856 (4.5) |  |
| Non-Hispanic White | 20,837 (75.1) | 44,294 (65.4) | 10,449 (55.4) | 69,665 (53.1) |  |
| Parent Education |  |  |  |  | <0.001 |
| No High School | 719 (2.6) | 1,409 (2.1) | 294 (1.6) | 3,444 (2.6) |  |
| Some High School | 4,005 (14.4) | 7,726 (11.4) | 2,063 (10.9) | 15,879 (12.1) |  |
| High School Degree | 9,017 (32.5) | 18,367 (27.1) | 4,871 (25.8) | 33,878 (25.8) |  |
| Some College | 9,235 (33.3) | 22,282 (32.9) | 6,157 (32.6) | 38,585 (29.4) |  |
| 4 Years College | 3,213 (11.6) | 11,269 (16.7) | 3,327 (17.6) | 22,892 (17.4) |  |
| >4 Years College | 1,481 (5.3) | 6,320 (9.3) | 2,062 (10.9) | 15,224 (11.6) |  |
| Missing | 91 (0.3) | 326 (0.5) | 99 (0.5) | 1,369 (1.0) |  |
| Insurance |  |  |  |  | <0.001 |
| Government | 15,639 (56.3) | 32,697 (48.3) | 9,585 (50.8) | 66,933 (51.0) |  |
| Private | 11,326 (40.8) | 32,581 (48.1) | 9,011 (47.8) | 62,020 (47.3) |  |
| Self-Pay | 406 (1.5) | 675 (1.0) | 105 (0.6) | 1,212 (0.9) |  |
| Other | 390 (1.4) | 1,746 (2.6) | 172 (0.9) | 1,106 (0.8) |  |
| Obstetric Comorbidity Score (Non-Transfusion), mean (SD) | 6.17 (9.08) | 7.01 (10.22) | 8.58 (12.06) | 11.34 (14.29) | <0.001 |
| Nulliparous | 11,998 (43.2) | 29,679 (43.8) | 7,972 (42.2) | 55,116 (42.0) | <0.001 |
| Cesarean birth | 7,430 (26.8) | 20,239 (29.9) | 6,340 (33.6) | 46,315 (35.3) | <0.001 |
| Obstetric patients with treatable complications | 27,761 (100.0) | 67,699 (100.0) | 18,873 (100.0) | 131,271 (100.0) |  |
| Infection | 13,501 (48.6) | 35,584 (52.6) | 10,490 (55.6) | 66,658 (50.8) | <0.001 |
| Hemorrhage | 14,823 (53.4) | 33,608 (49.6) | 8,815 (46.7) | 68,879 (52.5) | <0.001 |
| SMM (without blood product transfusion) | 634 (2.3) | 1,671 (2.5) | 650 (3.4) | 4,780 (3.6) | <0.001 |
| SMM (including blood product transfusion) | 2,604 (9.4) | 5,523 (8.2) | 1,592 (8.4) | 12,182 (9.3) | <0.001 |
| Birth Hospital Location^a^ |  |  |  |  | <0.001 |
| Metropolitan | 14,267 (51.4) | 63,202 (93.4) | 18,010 (95.4) | 131,271 (100.0) |  |
| Micropolitan | 10,011 (36.1) | 4,298 (6.4) | 863 (4.6) | 0 (0.0) |  |
| Noncore | 3,483 (12.6) | 199 (0.3) | 0 (0.0) | 0 (0.0) |  |
| Annual Obstetric Volume |  |  |  |  | <0.001 |
| 10-500 | 16,464 (59.3) | 868 (1.3) | 0 (0.0) | 0 (0.0) |  |
| 501-1000 | 10,874 (39.2) | 19,842 (29.3) | 0 (0.0) | 734 (0.6) |  |
| 1001-2000 | 423 (1.5) | 40,210 (59.4) | 7,985 (42.3) | 11,388 (8.7) |  |
| >2000 | 0 (0.0) | 6,779 (10.0) | 10,888 (57.7) | 119,149 (90.8) |  |
| Hospital Ownership |  |  |  |  | <0.001 |
| Government | 2,018 (7.3) | 2,094 (3.1) | 0 (0.0) | 14,146 (10.8) |  |
| Non-Profit | 22,942 (82.6) | 54,113 (79.9) | 16,459 (87.2) | 111,789 (85.2) |  |
| Profit | 2,782 (10.0) | 11,280 (16.7) | 2,414 (12.8) | 5,336 (4.1) |  |
| Missing | 19 (0.1) | 212 (0.3) | 0 (0.0) | 0 (0.0) |  |
| Teaching Status^b^ |  |  |  |  | <0.001 |
| Major-Teaching | 633 (2.3) | 2,534 (3.7) | 6,514 (34.5) | 74,692 (56.9) |  |
| Minor-Teaching | 8,205 (29.6) | 40,656 (60.1) | 9,296 (49.3) | 50,966 (38.8) |  |
| Non-Teaching | 18,900 (68.1) | 24,297 (35.9) | 3,063 (16.2) | 5,613 (4.3) |  |
| Missing | 23 (0.1) | 212 (0.3) | 0 (0.0) | 0 (0.0) |  |

Abbreviations: SD – Standard Deviation, SMM – Severe Maternal Morbidity

^a^Rurality is defined by Urban Influence Codes [UIC] with metropolitan: UIC 1, 2; micropolitan: UIC 3,5,8; non-core: UIC 4,6,7,9,10,11,12.

^b^Teaching status: Major teaching is defined by the American Hospital Association as being a member of the Council of Teaching Hospitals. Minor teaching is defined as those that are teaching hospitals but are not members and have at least one intern or resident.
