# Supplementary material for: Maternal levels of care and association with severe maternal morbidity during birth hospitalizations
Source: PLoS One. 2026 Jul 23;21(7):e0353016. doi: 10.1371/journal.pone.0353016 (PMC13395347; doi:10.1371/journal.pone.0353016)
Supplement: S4 Table — (DOCX) [file pone.0353016.s006.docx]

**S4 Table. Associations of levels of maternal care with SMM including blood product transfusion**

| **Among all obstetric patients** | | |
| --- | --- | --- |
| Level of maternal care | Unadjusted (RR, 95% CI) | Adjusted (aRR, 95% CI) |
| I | 0.75 (0.64, 0.88) | 1.15 (0.97, 1.36) |
| II | 0.72 (0.60, 0.85) | 1.07 (0.92, 1.23) |
| III | 0.77 (0.63, 0.93) | 1.00 (0.85, 1.18) |
| IV | Reference | Reference |
| **Among obstetric patients with common treatable childbirth conditions** | | |
| Level of maternal care | Unadjusted (RR, 95% CI) | Adjusted (aRR, 95% CI) |
| I | 1.01 (0.87, 1.17) | 1.28 (1.08, 1.52) |
| II | 0.88 (0.75, 1.03) | 1.13 (0.97, 1.31) |
| III | 0.91 (0.73, 1.13) | 1.09 (0.89, 1.35) |
| IV | Reference | Reference |
| **Among obstetric patients with infection** | | |
| Level of maternal care | Unadjusted (RR, 95% CI) | Adjusted (aRR, 95% CI) |
| I | 0.69 (0.58, 0.82) | 1.01 (0.85, 1.20) |
| II | 0.66 (0.56, 0.78) | 0.94 (0.82, 1.09) |
| III | 0.74 (0.61, 0.89) | 0.95 (0.79, 1.14) |
| IV | Reference | Reference |
| **Among obstetric patients with hemorrhage** | | |
| Level of maternal care | Unadjusted (RR, 95% CI) | Adjusted (aRR, 95% CI) |
| I | 1.08 (0.91, 1.28) | 1.34 (1.10, 1.64) |
| II | 0.97 (0.82, 1.15) | 1.18 (1.002, 1.40) |
| III | 1.03 (0.80, 1.31) | 1.14 (0.89, 1.45) |
| IV | Reference | Reference |

Abbreviations: SMM – Severe Maternal Morbidity, RR – Risk Ratio, aRR – adjusted Risk Ratio, CI – Confidence Interval

Model covariates include birth parent age, race and ethnicity, birth parent education, birth parent insurance type, obstetric comorbidity score, parity, birth hospital location, state, and year.
