# Supplementary material for: Maternal levels of care and association with severe maternal morbidity during birth hospitalizations
Source: PLoS One. 2026 Jul 23;21(7):e0353016. doi: 10.1371/journal.pone.0353016 (PMC13395347; doi:10.1371/journal.pone.0353016)
Supplement: S5 Table — (DOCX) [file pone.0353016.s007.docx]

**S5 Table. Supplemental analysis stratified by state^a^ reporting range of point estimates from the adjusted^b^ models examining the association of level of maternal care and SMM without blood product transfusion among 1) all obstetric patients, 2) those with common treatable childbirth complications, 3) those with infection, and 4) those with hemorrhage**

| Level | All obstetric patients | Obstetric patients with common treatable childbirth complications | Obstetric patients with infection | Obstetric patients with hemorrhage |
| --- | --- | --- | --- | --- |
| I | 0.81-0.91 | 0.93-1.36 | 0.83-1.25 | 0.97-1.42 |
| II | 0.84-1.01 | 0.87-1.14 | 0.83-0.98 | 0.91-1.29 |
| III | 0.83-1.15 | 1.11-1.24 | 1.09-1.62 | 1.20-1.55 |
| IV | Reference | Reference | Reference | Reference |

^a^State data cannot be individually reported per our data use agreement, thus the ranges reflect the point estimates for the four states analyzed.

^b^Model covariates include birth parent age, race and ethnicity, birth parent education, birth parent insurance type, obstetric comorbidity score, parity, birth hospital location, and year.
