# Supplementary material for: Maternal levels of care and association with severe maternal morbidity during birth hospitalizations
Source: PLoS One. 2026 Jul 23;21(7):e0353016. doi: 10.1371/journal.pone.0353016 (PMC13395347; doi:10.1371/journal.pone.0353016)
Supplement: S1 File — (DOCX) [file pone.0353016.s008.docx]

**S1 File. Full Model for association between level of maternal care and SMM without transfusion for all obstetric patients.**

---------------------------------------------------------------------------------

| Robust

SMM | IRR std. err. z P>|z| [95% conf. interval]

----------------+----------------------------------------------------------------

LOC_final_10 |

Level 1 | .8721533 .0608496 -1.96 0.050 .7606853 .9999555

Level 2 | .9235848 .0536092 -1.37 0.171 .8242692 1.034867

Level 3 | 1.021708 .0907193 0.24 0.809 .8585131 1.215924

Level 4 | 1 (base)

|

hosp_uic3 |

Metropolitan | 1 (base)

Micropolitan | 1.108312 .0801546 1.42 0.155 .9618378 1.277091

Noncore | .9996095 .1228187 -0.00 0.997 .7856807 1.271788

|

mage_cat |

<20 | .9819901 .0360244 -0.50 0.620 .9138622 1.055197

20-24 | .912651 .0188599 -4.42 0.000 .8764248 .9503746

25-34 | 1 (base)

35-39 | 1.175984 .0296078 6.44 0.000 1.119362 1.23547

40+ | 1.415238 .0545045 9.02 0.000 1.312343 1.5262

|

racem_eth |

White | 1 (base)

Black | 1.189032 .0416627 4.94 0.000 1.110116 1.273559

Hispanic | 1.104033 .0372704 2.93 0.003 1.033349 1.179552

Asian | 1.334762 .0591378 6.52 0.000 1.223745 1.455852

Other | 1.093096 .0458493 2.12 0.034 1.006828 1.186756

|

educatv2_M |

Missing | 1.163197 .1281932 1.37 0.170 .9372265 1.443651

No HS | 1.16102 .094075 1.84 0.065 .9905325 1.360852

Some HS | 1.035385 .0309264 1.16 0.244 .9765102 1.097809

HS Degree | 1.047614 .0221638 2.20 0.028 1.005062 1.091967

Some College | 1 (base)

4 Yr College | 1.000934 .0221514 0.04 0.966 .9584465 1.045306

>4 Yrs College | 1.047758 .0304286 1.61 0.108 .9897843 1.109127

|

insurance_mom |

Private | 1 (base)

Government | 1.02731 .0247233 1.12 0.263 .9799785 1.076928

SelfPay | .9558748 .0894192 -0.48 0.630 .7957445 1.148229

Other | 1.189907 .1514092 1.37 0.172 .9272618 1.526947

|

birthyear |

2010 | 1 (base)

2011 | .9954318 .0365413 -0.12 0.901 .9263281 1.069691

2012 | 1.035404 .042468 0.85 0.396 .9554261 1.122077

2013 | 1.022329 .0465141 0.49 0.627 .93511 1.117684

2014 | .9206219 .044615 -1.71 0.088 .8372025 1.012353

2015 | .967315 .049842 -0.64 0.519 .8743973 1.070107

2016 | .9180329 .0421145 -1.86 0.062 .839092 1.0044

2017 | .8279525 .0393862 -3.97 0.000 .7542464 .9088613

2018 | .8149884 .0370831 -4.50 0.000 .7454535 .8910095

2019 | .8051297 .0430172 -4.06 0.000 .7250818 .8940147

2020 | .8309277 .0445376 -3.46 0.001 .7480644 .9229699

|

state2 |

A | 1 (base)

B | .9769673 .0606839 -0.38 0.708 .8649839 1.103448

C | 1.023554 .0612267 0.39 0.697 .9103196 1.150874

D | 1.03209 .074769 0.44 0.663 .8954739 1.189549

|

cindx_ntscore | 1.059357 .000815 74.95 0.000 1.057761 1.060956

|

nulliparous |

0 | 1 (base)

1 | 1.183269 .0257895 7.72 0.000 1.133787 1.23491

|

_cons | .0033013 .0002396 -78.71 0.000 .0028635 .003806

---------------------------------------------------------------------------------
