# Supplementary material for: Maternal levels of care and association with severe maternal morbidity during birth hospitalizations
Source: PLoS One. 2026 Jul 23;21(7):e0353016. doi: 10.1371/journal.pone.0353016 (PMC13395347; doi:10.1371/journal.pone.0353016)
Supplement: S2 File — (DOCX) [file pone.0353016.s009.docx]

**S2 File. Full Model for association between level of maternal care and SMM without transfusion for obstetric patients with common treatable childbirth complications.**

---------------------------------------------------------------------------------

| Robust

SMM | IRR std. err. z P>|z| [95% conf. interval]

----------------+----------------------------------------------------------------

LOC_final_10 |

Level 1 | 1.051474 .079279 0.67 0.506 .9070262 1.218926

Level 2 | 1.028481 .0510338 0.57 0.571 .9331666 1.133531

Level 3 | 1.207045 .0917734 2.47 0.013 1.039933 1.401011

Level 4 | 1 (base)

|

hosp_uic3 |

Metropolitan | 1 (base)

Micropolitan | 1.094042 .0895374 1.10 0.272 .9319033 1.284391

Noncore | 1.159573 .1616683 1.06 0.288 .8823144 1.523959

|

mage_cat |

<20 | 1.032569 .0592753 0.56 0.577 .9226892 1.155535

20-24 | .8862347 .0331486 -3.23 0.001 .8235889 .9536456

25-34 | 1 (base)

35-39 | 1.188571 .0475514 4.32 0.000 1.098932 1.285522

40+ | 1.445555 .0818035 6.51 0.000 1.293795 1.615116

|

racem_eth |

White | 1 (base)

Black | 1.028264 .0443031 0.65 0.518 .9449967 1.118868

Hispanic | 1.176128 .0603254 3.16 0.002 1.063642 1.300511

Asian | 1.56664 .0832964 8.44 0.000 1.4116 1.738707

Other | 1.117274 .0625179 1.98 0.048 1.001221 1.246779

|

educatv2_M |

Missing | 1.141962 .1466433 1.03 0.301 .8878627 1.468782

No HS | 1.089023 .1310514 0.71 0.479 .8602104 1.378699

Some HS | 1.002944 .0431535 0.07 0.946 .9218327 1.091192

HS Degree | 1.051826 .0367671 1.45 0.148 .9821766 1.126414

Some College | 1 (base)

4 Yr College | 1.035769 .0403942 0.90 0.368 .959548 1.118045

>4 Yrs College | 1.036824 .047432 0.79 0.429 .9479046 1.134084

|

insurance_mom |

Private | 1 (base)

Government | .8989941 .0327652 -2.92 0.003 .8370156 .965562

SelfPay | .8992725 .087737 -1.09 0.277 .7427529 1.088775

Other | .8744676 .0839616 -1.40 0.162 .7244627 1.055532

|

birthyear |

2010 | 1 (base)

2011 | .9465317 .0510751 -1.02 0.309 .8515381 1.052122

2012 | .9553605 .051126 -0.85 0.393 .8602315 1.061009

2013 | .9165535 .0569399 -1.40 0.161 .8114802 1.035232

2014 | .911379 .0547202 -1.55 0.122 .8101995 1.025194

2015 | .8754277 .0565266 -2.06 0.039 .7713615 .9935338

2016 | .9307937 .0593782 -1.12 0.261 .8213962 1.054761

2017 | .9146618 .0599879 -1.36 0.174 .8043306 1.040127

2018 | .9137044 .0495671 -1.66 0.096 .8215412 1.016207

2019 | .8921894 .052093 -1.95 0.051 .7957145 1.000361

2020 | .8893792 .055136 -1.89 0.059 .7876218 1.004283

|

state2 |

A | 1 (base)

B | .8846293 .0457883 -2.37 0.018 .7992879 .9790828

C | 1.06424 .0525178 1.26 0.207 .9661285 1.172316

D | 1.09086 .0616324 1.54 0.124 .9765111 1.2186

|

cindx_ntscore | 1.046952 .0008555 56.15 0.000 1.045277 1.04863

|

nulliparous |

0 | 1 (base)

1 | .9820719 .0305447 -0.58 0.561 .9239936 1.043801

|

_cons | .0148255 .0009932 -62.86 0.000 .0130012 .0169058

---------------------------------------------------------------------------------
