# Supplementary material for: Maternal levels of care and association with severe maternal morbidity during birth hospitalizations
Source: PLoS One. 2026 Jul 23;21(7):e0353016. doi: 10.1371/journal.pone.0353016 (PMC13395347; doi:10.1371/journal.pone.0353016)
Supplement: S3 File — (DOCX) [file pone.0353016.s010.docx]

**S3 File. Full Model for association between level of maternal care and SMM without transfusion for obstetric patients with infection.**

---------------------------------------------------------------------------------

| Robust

SMM | IRR std. err. z P>|z| [95% conf. interval]

----------------+----------------------------------------------------------------

LOC_final_10 |

Level 1 | .9013209 .0849219 -1.10 0.270 .7493414 1.084125

Level 2 | .8469484 .0589358 -2.39 0.017 .7389673 .9707082

Level 3 | 1.148103 .1128844 1.40 0.160 .9468659 1.392108

Level 4 | 1 (base)

|

hosp_uic3 |

Metropolitan | 1 (base)

Micropolitan | 1.155615 .1141045 1.46 0.143 .9522834 1.402362

Noncore | 1.08221 .2313112 0.37 0.712 .7118296 1.645307

|

mage_cat |

<20 | 1.343147 .1054493 3.76 0.000 1.151587 1.566573

20-24 | 1.005079 .0568305 0.09 0.929 .8996431 1.122871

25-34 | 1 (base)

35-39 | .9970553 .0687596 -0.04 0.966 .8709999 1.141354

40+ | 1.058418 .1093308 0.55 0.583 .8644326 1.295935

|

racem_eth |

White | 1 (base)

Black | 1.048162 .0645166 0.76 0.445 .9290418 1.182556

Hispanic | 1.184333 .1029457 1.95 0.052 .9988142 1.404309

Asian | 1.503259 .155606 3.94 0.000 1.227224 1.841381

Other | 1.138462 .1038951 1.42 0.155 .952004 1.36144

|

educatv2_M |

Missing | 1.191223 .225007 0.93 0.354 .8226433 1.724941

No HS | .9626798 .1855206 -0.20 0.844 .659849 1.404492

Some HS | .9615742 .0554 -0.68 0.496 .8588985 1.076524

HS Degree | 1.063002 .0454876 1.43 0.153 .9774848 1.156002

Some College | 1 (base)

4 Yr College | .9957302 .0722635 -0.06 0.953 .8637083 1.147932

>4 Yrs College | .9242336 .0781259 -0.93 0.351 .7831217 1.090773

|

insurance_mom |

Private | 1 (base)

Government | 1.013522 .0532937 0.26 0.798 .9142707 1.123548

SelfPay | 1.189117 .2045652 1.01 0.314 .8487724 1.665934

Other | .9009436 .1483557 -0.63 0.526 .6524283 1.24412

|

birthyear |

2010 | 1 (base)

2011 | .8592557 .0687429 -1.90 0.058 .7345542 1.005127

2012 | .8600774 .071974 -1.80 0.072 .7299721 1.013372

2013 | .7796979 .0642962 -3.02 0.003 .6633362 .9164715

2014 | .8240696 .0667173 -2.39 0.017 .7031532 .9657791

2015 | .7672065 .0675371 -3.01 0.003 .6456261 .9116821

2016 | .8595545 .0933367 -1.39 0.163 .6947743 1.063416

2017 | .9280415 .0821933 -0.84 0.399 .7801526 1.103965

2018 | .9529094 .0787492 -0.58 0.559 .8104153 1.120458

2019 | .8624111 .0798664 -1.60 0.110 .7192602 1.034053

2020 | 1.038254 .0947695 0.41 0.681 .8681765 1.241651

|

state2 |

A | 1 (base)

B | .8338847 .063464 -2.39 0.017 .7183301 .9680279

C | .8963975 .0584393 -1.68 0.093 .7888743 1.018576

D | .9637787 .0728039 -0.49 0.625 .8311465 1.117576

|

cindx_ntscore | 1.047041 .0010561 45.57 0.000 1.044973 1.049113

|

nulliparous |

0 | 1 (base)

1 | 1.294112 .0554296 6.02 0.000 1.189908 1.407443

|

_cons | .0121051 .001057 -50.55 0.000 .010201 .0143646

---------------------------------------------------------------------------------
