# Supplementary material for: Maternal levels of care and association with severe maternal morbidity during birth hospitalizations
Source: PLoS One. 2026 Jul 23;21(7):e0353016. doi: 10.1371/journal.pone.0353016 (PMC13395347; doi:10.1371/journal.pone.0353016)
Supplement: S4 File — (DOCX) [file pone.0353016.s011.docx]

**S4 File. Full Model for association between level of maternal care and SMM without transfusion for obstetric patients with hemorrhage.**

---------------------------------------------------------------------------------

| Robust

SMM | IRR std. err. z P>|z| [95% conf. interval]

----------------+----------------------------------------------------------------

LOC_final_10 |

Level 1 | 1.187325 .1081838 1.88 0.060 .9931436 1.419474

Level 2 | 1.165531 .0653479 2.73 0.006 1.044238 1.300913

Level 3 | 1.244155 .1011166 2.69 0.007 1.060949 1.458997

Level 4 | 1 (base)

|

hosp_uic3 |

Metropolitan | 1 (base)

Micropolitan | 1.039104 .1018417 0.39 0.696 .8574989 1.259171

Noncore | 1.131794 .1729108 0.81 0.418 .8389277 1.5269

|

mage_cat |

<20 | .7210877 .0631559 -3.73 0.000 .6073461 .8561304

20-24 | .7510732 .0325666 -6.60 0.000 .6898808 .8176933

25-34 | 1 (base)

35-39 | 1.283096 .0585487 5.46 0.000 1.173325 1.403137

40+ | 1.581316 .0891166 8.13 0.000 1.415951 1.765993

|

racem_eth |

White | 1 (base)

Black | 1.068619 .0459486 1.54 0.123 .982252 1.16258

Hispanic | 1.065857 .0598519 1.14 0.256 .9547739 1.189863

Asian | 1.567068 .0838371 8.40 0.000 1.411072 1.74031

Other | 1.089631 .0732422 1.28 0.202 .9551331 1.243069

|

educatv2_M |

Missing | 1.280645 .2003078 1.58 0.114 .9425205 1.740069

No HS | 1.168721 .0899918 2.02 0.043 1.005004 1.359107

Some HS | 1.080895 .0662702 1.27 0.205 .9585088 1.218909

HS Degree | 1.02754 .0463048 0.60 0.547 .9406765 1.122424

Some College | 1 (base)

4 Yr College | .9885325 .0409058 -0.28 0.780 .9115238 1.072047

>4 Yrs College | .9932698 .0514541 -0.13 0.896 .8973723 1.099415

|

insurance_mom |

Private | 1 (base)

Government | .9139365 .0350485 -2.35 0.019 .8477608 .9852778

SelfPay | .7260374 .0938849 -2.48 0.013 .5634935 .9354682

Other | .9358221 .1186455 -0.52 0.601 .7299215 1.199804

|

birthyear |

2010 | 1 (base)

2011 | 1.0441 .0776947 0.58 0.562 .9024052 1.208044

2012 | 1.074477 .0731251 1.06 0.291 .9403025 1.227798

2013 | 1.080154 .0801564 1.04 0.299 .9339407 1.249257

2014 | 1.040228 .0781153 0.53 0.599 .8978591 1.205172

2015 | 1.012913 .0826223 0.16 0.875 .8632579 1.188513

2016 | 1.00323 .0791338 0.04 0.967 .8595249 1.170961

2017 | .9307695 .0827859 -0.81 0.420 .7818678 1.108029

2018 | .9204334 .0599179 -1.27 0.203 .8101796 1.045691

2019 | .9018441 .0692036 -1.35 0.178 .7759146 1.048212

2020 | .8562101 .0674801 -1.97 0.049 .7336601 .9992307

|

state2 |

A | 1 (base)

B | .9124676 .0599704 -1.39 0.163 .8021833 1.037914

C | 1.235093 .0692612 3.77 0.000 1.106538 1.378584

D | 1.260026 .0804884 3.62 0.000 1.111748 1.428082

|

cindx_ntscore | 1.045065 .0009215 49.99 0.000 1.04326 1.046872

|

nulliparous |

0 | 1 (base)

1 | .8567876 .0315537 -4.20 0.000 .7971227 .9209183

|

_cons | .0176284 .0017414 -40.88 0.000 .0145254 .0213942

---------------------------------------------------------------------------------
